# A Functional Bread Fermented with *Saccharomyces cerevisiae* UFMG A-905 Prevents Allergic Asthma in Mice

**DOI:** 10.1016/j.cdnut.2024.102142

**Published:** 2024-03-22

**Authors:** Ana Paula Carvalho Thiers Calazans, Thamires Melchiades Silva Milani, Ana Silvia Prata, Maria Teresa Pedrosa Silva Clerici, Jacques Robert Nicoli, Flaviano Santos Martins, Marcos Carvalho Borges

**Affiliations:** 1 Department of Internal Medicine, Ribeirao Preto Medical School, University of Sao Paulo, Ribeirao Preto, Brazil; 2 Department of Food Engineering and Technology, School of Food Engineering, University of Campinas, Campinas, Brazil; 3 Department of Food Science and Nutrition, School of Food Engineering, University of Campinas, Campinas, Brazil; 4 Department of Microbiology, Institute of Biological Sciences, Federal University of Minas Gerais, Belo Horizonte, Brazil

**Keywords:** probiotic, long fermentation, yeast, hypersensitivity, primary prevention, *Saccharomyces cerevisiae* UFMG A-905

## Abstract

**Background:**

The administration of probiotics has been shown to be beneficial in asthma. The administration of *Saccharomyces cerevisiae* UFMG A-905 prevented asthma development. Traditionally, probiotics are administered using dairy-based matrices, but other vehicles (e.g., fruit juices, biscuits, candies, and breads) can be used.

**Objectives:**

This study aimed to assess the effect of bread fermented with *S. cerevisiae* UFMG A-905 in asthma prevention.

**Methods:**

Three breads were produced: fermented with commercial yeast, fermented with *S. cerevisiae* UFMG A-905, and fermented with *S. cerevisiae* UFMG A-905 with the addition of alginate microcapsules containing live *S. cerevisiae* UFMG A-905. Characterization of the microbial composition of the breads was performed. Male Balb/c mice were sensitized and challenged with ovalbumin. Breads were administered 10 d before the first sensitization and during sensitization and challenge protocol. Yeast fecal count, in vivo airway hyperresponsiveness, and airway and lung inflammation were assessed.

**Results:**

In UFMG A-905 bread, there was an increase in yeast number and a decrease in total and lactic acid bacteria. Animals that received *S. cerevisiae* UFMG A-905 fermented bread with microcapsules had a significant increase in yeast recovery from feces. *S. cerevisiae* UFMG A-905–fermented breads partially reduced airway inflammation, decreasing eosinophils and IL5 and IL13 concentrations. When adding microcapsules, the bread also diminished airway hyperresponsiveness and increased IL17A concentrations.

**Conclusions:**

*S. cerevisiae* UFMG A-905 was able to generate long-fermentation breads. Microcapsules were a safe and viable way to inoculate the live yeast into food. The administration of breads fermented with *S. cerevisiae* UFMG A-905 prevented asthma-like characteristics, being more pronounced when the breads contained microcapsules with live yeast.

## Introduction

Asthma is a heterogeneous and complex disease characterized by airway inflammation, hyperresponsiveness, and remodeling and is one of the most common diseases in the world [1,2]. The prevalence of asthma is increasing, especially in high-income and more urbanized countries [1]. Several factors are associated with this increased prevalence, such as changes in lifestyle, environmental exposures (e.g., indoor dampness, molds, and smoke exposure), obesity, diet, and gut microbiota [1,3]. A better understanding of the risk factors may lead to the development of novel strategies to prevent asthma.

Several preclinical studies have shown that bacteria, fungi, and other microbes may prevent asthma development, confirming the role of microbiota in the pathogenesis of allergic diseases [[4], [5], [6]]. Although these studies suggest a potential role of probiotics in preventing or treating allergic diseases, it is still necessary to evaluate the best microorganism, preparation, dose, and regime, and confirm their effects in clinical trials [7].

The microorganisms most commonly evaluated as probiotics are bacteria belonging to the genera *Lactobacillus* and *Bifidobacterium*, although other bacteria and yeasts are also used. *Saccharomyces cerevisiae* UFMG A-905, isolated from a Brazilian sugarcane-distilled alcoholic beverage, has probiotic characteristics and has prevented bacterial infections, experimental colitis and mucositis, food allergy, and asthma [6,[8], [9], [10], [11], [12], [13]]. *S. cerevisiae* UFMG A-905 acts on cell signal transduction, pathogenic bacteria cell adherence, and local and systemic immunomodulation [11,14,15]. We have shown that the administration of *S. cerevisiae* UFMG A-905 prevented the development of asthma-like characteristics in an animal model in a dose-dependent manner [6,16].

Probiotics are defined as “live microorganisms which when administered in adequate amounts confer a health benefit on the host” [17]. In this context, their therapeutic usage can have limitations and is influenced by factors such as water activity, acidity, oxygen concentration, temperature, shelf life, and interactions with other microorganisms in the products [18]. Despite the definition linking the beneficial action of probiotics to their cell number and viability, the use of nonviable microorganisms or their products (e.g., postbiotics, psychobiotics, nutribiotics, and gerobiotics) has also become a viable approach [18]. Traditionally, probiotics are administered using dairy-based products (yogurt, milk, kefir, ice cream, butter, cheese, and others). However, various factors, such as milk protein allergy, may require the use of other vehicles, such as fruit juices, biscuits, candies, and breads. As *S. cerevisiae* is widely used to ferment foods, the probiotic yeast could produce a functional bread whose consumption could prevent the development of asthma. Furthermore, considering the high temperature reached during baking, the addition of encapsulated yeast can help maintain viable probiotic cells in the bread [19].

Sourdough fermentation has important effects on bread characteristics such as rheology, sensory, and shelf life. It may produce functional foods changing the glycemic response, mineral bioavailability, gluten content, satiety, or gastrointestinal comfort [20,21]. However, due to a lack of standardization of some breads (e.g., microbial composition, fermentation process, type of flour), it is difficult to attribute a possible clinical effect to sourdough fermentation [20]. Thus, additional studies with specific strains, more standardized processes, and specific health clinical outcomes are still needed.

The present study aimed to evaluate the effect of bread fermented with *S. cerevisiae* UFMG A-905, with or without the addition of microcapsules containing the same yeast, in the prevention of asthma in an animal model.

## Material and Methods

### Probiotic yeast

The yeast *S. cerevisiae* UFMG 905 was given by Prof. Dr. Carlos Augusto Rosa, from the Laboratório de Ecologia e Biotecnologia de Leveduras, Departamento de Microbiologia/Instituto de Ciências Biológicas, Universidade Federal de Minas Gerais. The strain was identified and classified as *S. cerevisiae* using the YEASTCOMPARE program (Cirriello CJ and Lachance MA. YEASTCOMPARE. London, ON, Canada). The yeast was preserved in a medium (1% yeast extract, 2% peptone, and 20% glycerol) at −80°C. For all experiments, yeast was grown in yeast extract peptone glucose (YPG) medium (1% yeast extract, 2% peptone, and 2% glucose) at 37°C for 24 h. Then, the yeast culture was concentrated to obtain a concentration of 10^9^ number of colony-forming units (cfu/ml).

### Microcapsules production

Microcapsules with *S. cerevisiae* UFMG A-905 were developed using the ionotropic gelation method, as previously described [22]. Shortly, a 2% w/w sodium alginate suspension (Dinâmica Química Contemponânea Ltda.) was prepared and *S. cerevisiae* UFMG A-905 at a concentration of 10^9^ cfu/ml was added. The mixture was pumped through a jacketed double-fluid atomizer nozzle (I.D. 1 mm) into a 0.1 M calcium chloride bath. The capsules recovered were then lyophilized for 24 h (30°C with 160 mmHg pressure) for later use.

The number of viable cells inside the microcapsules was determined by solubilizing 1 g of beads in 9 mL of citrate–phosphate buffer (pH 7.0) and cultivating serial decimal dilutions onto potato dextrose agar medium. After 72 h at 37°C, cell counts were determined and expressed as the cfu/g of microcapsule.

### Bread preparation

Three bread formulations were produced: bread fermented with commercial yeast (COM bread), bread fermented with the probiotic yeast *S. cerevisiae* UFMG A-905 (UFMG-A905 bread), and bread fermented with *S. cerevisiae* UFMG A-905 with the addition of microcapsules containing viable *S. cerevisiae* UFMG A-905 (UFMG-A905-C bread) (Brazilian patent registration no. BR1020210266465).

The preparation of breads was carried out in 3 stages: the production of acid mass, sponge, and the final dough. For the acid mass production, 50% wheat flour (Anaconda and Moinho Curitiba) and 50% boiled and filtered water were used.

In the UFMG-A905 and UFMG-A905-C breads, 2 mL of *S. cerevisiae* UFMG A-905 at 10^9^ cfu/ml were added in 100 g of acid mass. For the COM bread, 3.2 g of instant dry biological yeast (Fleischmann, AB Brasil Indústria e Comércio de Alimentos LTDA) containing commercial *S. cerevisiae* were added to 100 g of acid mass. Acid masses were kept at 14°C in a biochemical oxygen demand incubator (Marconi, MA 415) for 15 d, and twice a day, 200 g of flour and 200 mL of boiled and filtered water were added.

For the production of the sponge, 40% acid mass, 45% wheat flour, and 11.6% water were used. After 4 h of fermentation, the following ingredients were added: 35% wheat flour, 40% vegetable fat, 40% sugar, 18% salt, and 9.8% water. The loaves were molded, fermented for another 10 min, and baked.

In order to have better acceptance by the animals, the commercial and UFMG-A905 breads were lyophilized and reconstituted into biscuits. For this, 4% of calcium lignosulfanate (Lignobond DD, Lignotech Brasil Produtos de lignina LTDA) and 70% of water were used. These ingredients were homogenized, molded, and dried in an oven.

Additionally, to reconstituted yeast bread biscuits, microcapsules were added at a rate of 10%. Thus, the animals that received the biscuit added with microcapsules received a dose of 9.5 × 10^6^ cfu/g of biscuit.

All the biscuits were packed in sealed polyethylene bags and offered in a dose of 2.5 ± 0.2 g per mouse.

### Characterization of microbial composition

Samples of 25 g were collected from the acid masses, loaves, and breads, and cultured, in triplicate, onto de Man, Rogosa, and Sharpe Agar, potato dextrose agar, and standard counting agar medium to assess the number of lactic acid bacteria, yeast, and total bacteria, respectively. After 72 h of anaerobic incubation at 37°C, cell counts were determined and expressed as the number of cfu/g.

### Animals

**FIGURE 1:**
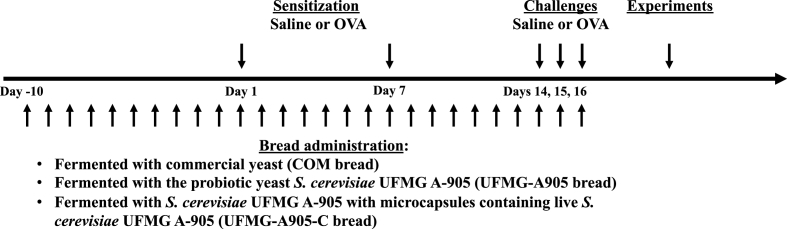
Protocol of allergic sensitization, challenge, and administration of breads. Mice were sensitized intraperitoneally twice with ovalbumin (OVA) and adjuvant aluminum hydroxide with an interval of 7 d. After 1 wk, they were challenged with OVA for 3 consecutive days. Breads were administered 10 d before the first sensitization and during sensitization and challenges. COM bread, bread fermented with commercial yeast; UFMG-A905 bread, bread fermented with the probiotic yeast *Saccharomyces cerevisiae* UFMG A-905; UFMG-A905-C bread, bread fermented with *Saccharomyces cerevisiae* UFMG A-905 with microcapsules containing live *S. cerevisiae* UFMG A-905.

Specific pathogen-free male Balb/c mice, 6 to 8 wk old, were used. The animals were obtained from the breeding facility of Ribeirão Preto Medical School, Ribeirão Preto, São Paulo, Brazil, and kept in separate autoclaved cages according to the treatment protocol (Figure 1). Water and food were autoclaved weekly and provided ad libitum. Experiments were approved by the Ethics Committee on Animal Use of Ribeirão Preto Medical School (protocol 023/2016) and carried out in accordance with the National Research Council's Guide for the Care and Use of Laboratory Animals.

### Allergen sensitization and challenges

The mice were sensitized intraperitoneally (I.P.) twice with 10 μg ovalbumin (OVA, Sigma grade V, Sigma–Aldrich) and 1 mg of Al(OH)3 (Sigma–Aldrich) 7 d apart. One week later, they were challenged intranasally with the instillation of 50 μL of OVA (10 μg) under light anesthesia for 3 consecutive days.

### Experimental design

Animals were divided into the following 5 groups (Figure 1): saline-treated and saline-challenged (SAL group); saline-treated and OVA-challenged (OVA group); fed bread fermented with commercial yeast and OVA-challenged (COM bread); fed bread fermented with *S. cerevisiae* UFMG A-905 and OVA-challenged (UFMG-A905 bread); and fed bread fermented with *S. cerevisiae* UFMG A-905 and supplemented with microcapsules containing live *S. cerevisiae* UFMG A-905 and OVA-challenged (UFMG-A905-C bread). Bread administration started 10 d before the first sensitization and continued during sensitization and challenge protocol (a total of 27 d). Biscuit consumption was monitored daily.

### Animal weight and yeast fecal count

Mouse weight was evaluated weekly. To evaluate fecal concentrations of yeast, freshly evacuated feces were collected on the first day of bread administration, the first day of sensitization, and the day of the challenge. Then, fecal samples were weighed, homogenized, and submitted to decimal serial dilutions in phosphate-buffered saline. Ten microliters of the dilutions were plated, in triplicate, onto Sabouraud dextrose agar supplemented with 100 mg/L of chloramphenicol and incubated at 37°C for 72 h for a count of the yeast that was expressed as cfu/g of feces.

### Measurement of in vivo respiratory function

Measurements of respiratory parameters were performed as previously described [6,16,23]. Briefly, 24 h after the last challenge, the animals were anesthetized I.P. with xylazine and ketamine and connected to a mechanical ventilator for small animals (FlexiVent, Scireq) under a respiratory rate of 150/min and positive end-expiratory pressure of 3 cm H_2_O. They were paralyzed with pancuronium bromide I.P. and respiratory measurements were made at baseline and after increasing concentrations of methacholine (6.25, 12.5, 25, and 50 mg/mL). Airway responsiveness was expressed as total resistance (RRS), total elastance (ERS), tissue resistance (G), and tissue elastance (H) and was determined from curves with a coefficient of determination ≥0.85.

### Collection of bronchoalveolar lavage and cell counting

After airway hyperresponsiveness evaluation, bronchoalveolar lavage (BAL) was collected as previously described [6,16,23]. BAL supernatants were stored at −80°C for analysis of cytokine production. The total cell number was counted using the Neubauer chamber and trypan blue. Then, BAL samples were centrifuged in a Cytospin centrifuge (Cytospin IV, Thermo Scientific) and stained with the quick Panotic kit (Laborclin). Differential cell number was evaluated by counting 300 inflammatory cells per slide.

### Cytokine concentrations in BAL and lung homogenate

After BAL collection, the chest was opened, blood was washed from the lungs, and the right lung was stored in RNAlater (Qiagen) at −80°C. Then, 50 mg of lung tissue was homogenized with a protease inhibitor cocktail (Complete EDTA-free, Roche). The supernatant was collected and stored at −80°C for analysis of cytokine production. The concentrations of IL5 were measured in the BAL and IL4, IL5, IL13, IL17A, and IL10 in the lung homogenate using the ELISA method, according to the manufacturer’s instructions. IL4, IL5, and IL10 were detected by BD OptEIA set (BD Bioscience Pharmigen) and IL13 and IL17A by Ready-SET–GO! (eBioscience).

### Statistical analysis

The data were presented as mean and standard error and analyzed using GraphPad Prism software version 5.0 (GraphPad – Prism Software Inc.). One-way or 2-way analysis of variance was followed by Bonferroni post hoc test. Differences were considered statistically significant at *P* < 0.05.

## Results

### Microbial composition of acid masses, loaves, and breads

The acid mass of COM bread contained 5.7 × 10^6^ cfu of yeast/g, 1.32 × 10^8^ cfu of total bacteria/g, and 1.05 × 10^9^ cfu of lactic acid bacteria/g. In the acid mass made with *S. cerevisiae* UFMG A-905, there was an increase in the number of yeast and total bacteria and a decrease in lactic acid bacteria count (1.6 × 10^7^ cfu/g, 5.9 × 10^8^ cfu/g, and 4.9 × 10^8^ cfu/g, respectively).

After the final fermentation, in the COM bread, there were 6.85 × 10^4^ cfu of yeast/g, 1.2 × 10^9^ cfu of total bacteria/g, and 4.6 × 10^11^ cfu of lactic acid bacteria/g. In the UFMG A-905 bread, there was an increase in the number of yeast and a decrease in total bacteria and lactic acid bacteria (4.15 × 10^6^ cfu/g, 1.1 × 10^8^ cfu/g, and 3.4 × 10^8^ cfu/g, respectively).

There was no growth of microorganisms in the breads after baking.

### Mouse weight and yeast fecal count

**FIGURE 2:**
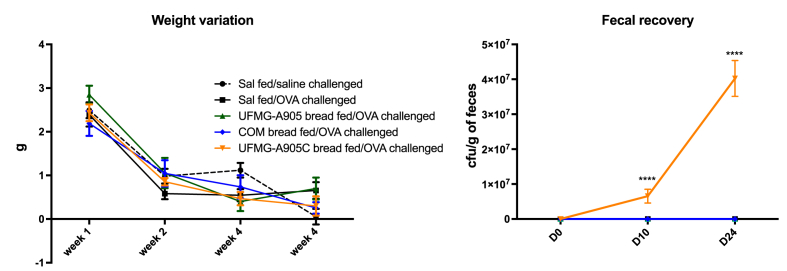
Effect of bread administration on animal’s weight and yeast fecal count. The mice’s weight was evaluated weekly. Samples of the animals' feces were collected on the first day of bread administration, the first day of sensitization, and the day of challenge in order to evaluate yeast excretion. COM bread, bread fermented with commercial yeast; UFMG-A905 bread, bread fermented with the probiotic yeast *Saccharomyces cerevisiae* UFMG A-905; UFMG-A905-C bread, bread fermented with *Saccharomyces cerevisiae* UFMG A-905 with microcapsules containing live *S. cerevisiae* UFMG A-905. Values are shown as mean ± SEM (*n* = 9–11 for animal’s weight and 3–5 for fecal recovery). ∗∗∗∗*P* < 0.0001. cfu: colony-forming units; OVA, ovalbumin; Sal, saline.

During the treatment protocol, sensitization, and challenges, the weight variation of the animals was not statistically different among the groups (Figure 2). On the first treatment day, there was no significant yeast growth in the feces of any group. On the first day of immunization and challenge, only animals that received UFMG-A905-C bread had a significant increase in yeast recovery (6.5 × 10^6^ ± 1.9 × 10^6^ cfu/g of feces and 4.0 × 10^7^ ± 5.1 × 10^6^ cfu/g of feces, respectively) (Figure 2).

### Hyperresponsiveness

**FIGURE 3:**
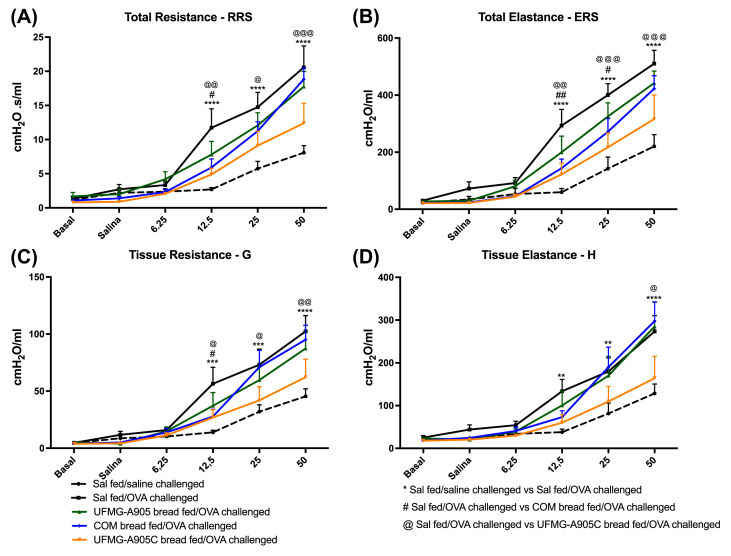
Effect of bread administration on airway hyperresponsiveness. Airway responsiveness was measured in response to increasing methacholine concentration, following sensitization and challenge. COM bread, bread fermented with commercial yeast; UFMG-A905 bread, bread fermented with the probiotic yeast *Saccharomyces cerevisiae* UFMG A-905; UFMG-A905-C bread, bread fermented with *Saccharomyces cerevisiae* UFMG A-905 with microcapsules containing live *S. cerevisiae* UFMG A-905. (A) Total resistance (RRS), (B) total elastance (ERS), (C) tissue resistance (G), and (D) tissue elastance (H). Values are shown as mean ± SEM (*n* = 7–11). ∗*P* < 0.05; ∗∗*P* < 0.01; ∗∗∗*P* < 0.001; ∗∗∗∗*P* < 0.0001. ∗Indicates Sal fed/saline-challenged compared with Sal fed/OVA-challenged; #Indicates Sal fed/OVA-challenged compared with COM bread fed/OVA-challenged; @Indicates Sal fed/OVA-challenged compared with UFMG-A905C bread fed/OVA-challenged. OVA, ovalbumin; Sal, saline.

Respiratory parameters are shown in Figure 3. OVA-challenged mice had a significant increase in airway hyperresponsiveness compared with saline-challenged mice (*P* < 0.001). The administration of bread fermented with *S. cerevisiae* UFMG A-905 with microcapsules signiﬁcantly decreased airway hyperresponsiveness, measured by RRS (*P* < 0.001), ERS (*P* < 0.05), G (*P* < 0.01), and H (*P* < 0.05) when compared with OVA-challenged mice. The administration of COM bread was also able to significantly reduce some parameters of airway hyperresponsiveness when compared with OVA-challenged mice (RRS [*P* < 0.05], ERS [*P* < 0.01]). The group that received the UFMG A-905 bread had no significant changes in hyperresponsiveness.

### Airway inflammation

**FIGURE 4:**
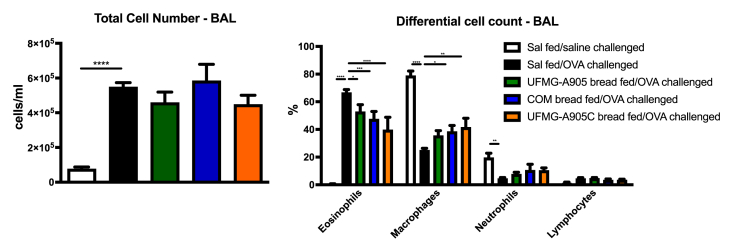
Effect of bread administration on total and differential cell counts (eosinophils, macrophages, lymphocytes, and neutrophils) in bronchoalveolar lavage (BAL). COM bread, bread fermented with commercial yeast; UFMG-A905 bread, bread fermented with the probiotic yeast *Saccharomyces cerevisiae* UFMG A-905; UFMG-A905-C bread, bread fermented with *Saccharomyces cerevisiae* UFMG A-905 with microcapsules containing live *S. cerevisiae* UFMG A-905. Values are shown as mean ± SEM (*n* = 6–11). ∗*P* < 0.05; ∗∗*P* < 0.01; ∗∗∗*P* < 0.001; ∗∗∗∗*P* < 0.0001. OVA, ovalbumin; Sal, saline.

Total and differential cell counts in BAL are shown in Figure 4. Total cell and eosinophil numbers were significantly increased in OVA-challenged mice compared with saline-challenged mice (*P* < 0.001). The administration of the 3 types of bread did not significantly decrease the total cell number. A significant reduction in the percentage of eosinophils in the BAL was observed in the UFMG A-905, UFMG-A905-C, and COM groups when compared with the OVA group (*P* < 0.05).

### Lung cytokines

**FIGURE 5:**
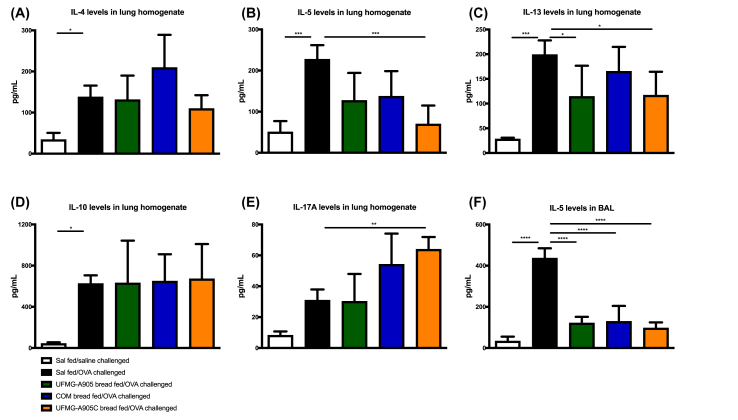
Effect of bread administration on cytokine concentrations in (A–E) lung homogenate and (F) bronchoalveolar lavage (BAL). COM bread, bread fermented with commercial yeast; UFMG-A905 bread, bread fermented with the probiotic yeast *Saccharomyces cerevisiae* UFMG A-905; UFMG-A905-C bread, bread fermented with *Saccharomyces cerevisiae* UFMG A-905 with microcapsules containing live *S. cerevisiae* UFMG A-905. Values are shown as mean ± SEM (*n* = 5–8). ∗*P* < 0.05; ∗∗*P* < 0.01; ∗∗∗*P* < 0.001; ∗∗∗∗*P* < 0.0001. OVA, ovalbumin; Sal, saline.

The concentrations of IL4, IL5, and IL13 were significantly increased (*P* < 0.05) in OVA-challenged mice compared with saline-challenged mice (Figure 5). The administration of UFMG A-905, UFMG-A905-C, and COM breads significantly reduced IL5 concentrations in the BAL (*P* < 0.001) when compared with OVA-challenged mice. In lung homogenate, when compared with OVA-challenged mice, the administration of UFMG-A905-C bread significantly decreased the concentrations of IL5 (*P* < 0.001) and IL13 (*P* < 0.05) and the UFMG-A905 bread significantly decreased IL5 concentrations (*P* < 0.05). The group that received the UFMG-A905-C had a significant increase in the IL17A concentrations (*P* < 0.05). The COM bread did not significantly change interleukin concentrations in lung homogenate.

## Discussion

A biotechnological challenge of using probiotics is how to administer them effectively and safely. Considering the widespread use of *S. cerevisiae* in the food industry, we evaluated the effects of fermented breads with *S. cerevisiae* UFMG A-905 on asthma prevention in an animal model. As the baking process kills yeast cells, we also evaluated the additional effect of viable probiotics by microencapsulating live *S. cerevisiae* UFMG A-905 with alginate. The development of breads fermented with *S. cerevisiae* UFMG A-905 partially reduced airway inflammation, as demonstrated by the reduction in eosinophils and IL5 and IL13 concentrations. When microcapsules containing live *S. cerevisiae* UFMG A-905 were added, the bread also diminished airway hyperresponsiveness and increased the concentrations of IL17A.

Bread is a widely accepted food and can be produced by several technologies. The use of sourdough for bread fermentation is a process developed in ancient Egyptian civilizations from the spontaneous fermentation of microorganisms presented in flour [21]. Sourdough bread fermentation produces different metabolites and more palatable breads, with better texture, volume, nutritional value, and longer shelf life [21]. Usually, its microbial composition is mainly of lactic acid bacteria (≥10^8^ cfu/g) and yeast (≤10^7^ cfu/g) [24], similar to the microbial composition of our bread fermented with commercial *S. cerevisiae*. The addition of *S. cerevisiae* UFMG A-905 in the process led to more significant yeast growth and less lactic acid bacteria.

Some studies have demonstrated the beneficial effects of sourdough bread on glycemic response, satiety, or gastrointestinal distress, whereas others have not [20]. A recent meta-analysis showed that sourdough bread reduced the increase of postprandial glycemia, especially if made with whole wheat flour [25]. However, it is challenging to attribute a possible clinical effect to its fermentation, especially if the sourdough bread lacks standardization [20]. We have developed a more standardized sourdough bread with specific fermentation conditions and yeast, *S. cerevisiae* UFMG A-905, which would be meaningful to the bakery industry.

Concomitantly to the sourdough fermentation with a specific yeast, we opted to add an emerging technology in the bread, i.e., alginate microcapsules with live *S. cerevisiae* UFMG A-905. Microcapsules may improve probiotic viability and activity during its shelf life [26]. Encapsulation can also protect bioactive compounds and probiotics, improving their stability, survival, and bioavailability, contributing to the development of products with better qualities [27,28].

Previous studies have shown that microencapsulation provides the probiotics more resistance to digestion and better survival during the baking process without altering the flavor or texture of the bread [19,29]. Then, microencapsulation can create a microenvironment that protects the organism and promotes more significant acting at the site of action. As the objective of our work had not been to evaluate the protection of alginate microcapsules during baking, they were added directly to the bread. Then, to assess the effect on intestinal survival, we evaluated the presence of yeast in the mouse feces. Only the animals that received bread with microcapsules had a significant increase in the number of yeasts during the treatment protocol, confirming that the microcapsules allowed *S. cerevisiae* UFMG A-905 to reach functional concentrations in the intestines of the animals.

After we evaluated the administration of the 3 breads in an asthma model, and to our knowledge, no studies assessed the use of bread fermented with probiotic yeasts in asthma prevention. The administration of the bread fermented with *S. cerevisiae* UFMG A-905 prevented the inflammatory response, i.e., it decreased the percentage of eosinophils and IL5 concentrations in the BAL and IL13 concentrations in the lung homogenate. However, it did not reduce airway hyperresponsiveness. Although airway hyperresponsiveness and inflammation are central features of asthma, previous studies have demonstrated that they are not totally dependent [30].

As the baking process kills the yeast, the protective effects of *S. cerevisiae* UFMG A-905 fermented bread may be from its wall constituents, such as β-glucans or mannans, or some products from its fermentation. It is well known that β-glucan has a significant immunomodulatory effect in the treatment and prevention of allergic diseases [31,32]. In an animal model using OVA, β-glucan reduced the influx of eosinophils and the production of Th2 cytokines [31]. β-glucan also reduced IL4 and IL5 concentrations and increased IL12 concentrations in individuals with allergic rhinitis [33]. In a humanized transgenic mouse strain, mannan derived from *S. cerevisiae* prevented the development of inflammation, bronchial hyperresponsiveness, and airway smooth muscle hyperplasia/hypertrophy [34]. Sourdough fermentation can also generate bioactive amino acids and peptides, and some yeasts, such as *S. cerevisiae*, are able to produce short-chain fatty acids that can protect against allergic diseases [[35], [36], [37]]. Thus, some of these compounds may have also contributed to the effect of the *S. cerevisiae* UFMG A-905 fermented bread.

With the addition of microcapsules, the bread effects were more pronounced, with a significant reduction in airway hyperresponsiveness, percentage of eosinophils, and Th2 cytokines concentrations (IL5 and IL13), and an increase in the IL17A concentrations. These results were similar to previous studies that demonstrated the action of live *S. cerevisiae* UFMG A-905 on asthma prevention [6,16]. IL5 and IL13 are major cytokines involved in asthma pathogenesis, related to differentiation, production, maturation, and activation of eosinophils, activation of fibroblasts, increased mucus production, and airway hyperresponsiveness. Of note, we demonstrated an increase in IL17A concentrations, suggesting a possible shift to Th17 response. The role of IL17A remains controversial in asthma. Some studies indicate that increased IL17 concentrations are associated with severe asthma [38]. However, other studies have demonstrated a dual role for IL17, and the exogenous administration of IL17A reduced the recruitment of pulmonary eosinophils, bronchial hyperresponsiveness, and allergic response [39,40]. Thus, in our study, another factor that may have contributed to the prevention of asthma characteristics was the increase in IL17A, similar to the findings of Fonseca et al. [6].

Generoso et al. [10] demonstrated that the viable and heat-killed *S. cerevisiae* UFMG A-905 prevented bacterial translocation, but only the live yeast increased secretory IgA concentrations, reinforcing our findings that the beneficial effects were more pronounced with the viable yeast. In an animal model of food allergy, the anti-inflammatory effect of the *S. cerevisiae* UFMG A-905 was also dependent on the yeast viability [13]. A possible explanation is that the *S. cerevisiae* UFMG A-905 can act by modifying the gut microbiota. The existence of a gut–lung axis and the relationship between asthma and gut microbiota has already been reported [41,42]. Previous studies demonstrated that probiotics or sourdough can change the gut microbiota in humans and mice [[43], [44], [45], [46], [47]]. Thus, the administration of bread fermented with *S. cerevisiae* UFMG A-905 could have altered mice gut microbiota and, consequently, prevented asthma development.

Previous studies had already shown the therapeutic potential of *S. cerevisiae* UFMG A-905 in asthma, but none has used it as a leaven or incorporated it into foods. This work has some limitations. First, we did not evaluate the bread fermented with commercial yeast added with microcapsules. Second, we did not assess the survival of *S. cerevisiae* UFMG A-905 microcapsules after baking. Third, we assessed the effect of bread fermented with *S. cerevisiae* UFMG A-905 only in an animal model of asthma induced by OVA. Although it is a classical model of asthma, it would be important to evaluate it in another model with a different allergen or even in a clinical trial. Finally, we did not assess microcapsule shelf life.

In conclusion, *S. cerevisiae* UFMG A-905 was able to generate long-fermentation breads. Adding microcapsules with the live yeast was a safe and viable way to inoculate probiotics into food. The administration of breads fermented with *S. cerevisiae* UFMG A-905 in an animal model of asthma was able to prevent asthma-line characteristics, being more pronounced when the breads contained microcapsules with the live yeast.

## Author contributions

The authors’ responsibilities were as follows – all authors: contributed to the study’s conception and design; APCTC, TMSM, ASP, MTPSC, FSM, and MCB: performed material preparation, data collection, and analysis; APCTC, MCB: wrote the first draft of the manuscript; and all authors: commented on previous versions of the manuscript and read and approved the final manuscript.

## Conflict of interest

MCB reports that financial support was provided by the State of São Paulo Research Foundation. All authors patented the breads fermented with the probiotic yeast *Saccharomyces cerevisiae* UFMG A-905.

## Funding

This study was funded by the São Paulo Research Foundation (FAPESP) Grant #2010/20600-4. We would like to thank the Coordenação de Aperfeiçoamento de Pessoal de Nível Superior (CAPES) for financial support. The funding sources had no involvement in study design; in the collection, analysis, and interpretation of data; in the writing of the report; or in the decision to submit the article for publication.

## Data availability

The datasets generated during and/or analyzed during the current study are available from the corresponding author upon reasonable request.
